# Hydrothermal Preparation and High Electrochemical Performance of NiS Nanospheres as Anode for Lithium-Ion Batteries

**DOI:** 10.3389/fchem.2021.812274

**Published:** 2022-02-03

**Authors:** Lin-Hui Wang, Long-Long Ren, Yu-Feng Qin, Qiang Li

**Affiliations:** ^1^ College of Information Science and Engineering, Shandong Agricultural University, Taian, China; ^2^ College of Mechanical and Electronic Engineering, Shandong Agricultural University, Taian, China; ^3^ College of Physics, University-Industry Joint Center for Ocean Observation and Broadband Communication, Qingdao University, Qingdao, China

**Keywords:** anodes, lithium-ion batteries, hydrothermal method, NiS nanospheres, electrochemical performance

## Abstract

Nickel sulfide has been widely studied as an anode material for lithium-ion batteries due to its environmental friendliness, low cost, high conductivity, and high theoretical capacity. A simple hydrothermal method was used to prepare NiS nanospheres materials with the size in the range of 100–500 nm. The NiS nanospheres electrodes exhibited a high reversible capacity of 1402.3 mAh g^−1^ at 200 mA g^−1^ after 280 cycles and a strong rate capability of 814.8 mAh g^−1^ at 0.8 A g^−1^ and 1130.5 mAh g^−1^ when back to 0.1 A g^−1^. Excellent electrochemical properties and the simple preparation method of the NiS nanospheres make it possible to prepare NiS on a large scale as the anode of lithium-ion batteries.

## Introduction

With the gradual exhaustion of fossil energy and the resulting emission of carbon oxides, the development of new green and sustainable energy, such as wind energy and solar energy, presents a strong trend, which puts forward higher requirements for energy storage and conversion technology (Li S. et al., 2021). As new energy storage devices, lithium-ion batteries (LIBs) have been widely studied due to their long service life, no memory effect, high charging efficiency, and environmental friendliness (Hong and Song, 2018; Hou et al., 2020; Li W. et al., 2020; Teng et al., 2020; Zheng et al., 2020; Gao et al., 2021; Li H. et al., 2021; Li Z. et al., 2021; Wang L et al., 2021). Since LIBs were commercialized by Sony in 1991, graphite has been the main anode of LIBs for a long period (Park and Lee, 2019). Although graphite has high conductivity and good cycling stability, its poor rate performance and low theoretical capacity will limit its application and development in the future (Dong et al., 2019; Ren et al., 2021; Xia et al., 2021).

Nanomaterials have shown excellent physical and chemical properties in many fields due to their larger specific surface area and higher activity (Wang et al., 2014; Wang et al., 2017; Li H. et al., 2019; Zhou et al., 2019; Gu et al., 2021; Wang X et al., 2021; Zhao et al., 2021; Liang et al., 2022; Wang et al., 2022). Transition metal sulfide (TMS) nanomaterials have been extensively researched in the field of the anode materials of LIBs due to low redox potential, good conductivity, strong cycling stability, and high theoretical capacity (Zhao et al., 2018; Wang et al., 2020; Yang et al., 2021). Among them, NiS is an excellent choice to replace graphite anodes because of its good stability, high theoretical capacity (590 mAh g^−1^), and high conductivity (Ren et al., 2021). Recently, Lee et al*.* synthesized hierarchical carbon-coated NiS with a discharge capacity of 606 mAh g^−1^ after 100 cycles (Park and Lee, 2019). Gao et al*.* prepared porous NiS@NSC tubules using biological templates, which showed a discharge capacity of 715.9 mAh g^−1^ at the 200th cycle (Dong et al., 2019). Wang et al. prepared NiS/C nanomaterials with biomass biochar, exhibiting a reversible capacity of 411.6 mAh g^−1^ at the 100th cycle (Xia et al., 2021). However, most NiS nanomaterials have not shown the satisfying reversible capacity and cycle stability, and complex preparations restricted its mass production.

In this work, NiS nanospheres were prepared by a hydrothermal method and showed outstanding performance as anodes for LIBs. The initial discharge and charge capacities reached 1418.5 mAh g^−1^ and 778.3 mAh g^−1^ at 0.2 A g^−1^, respectively. The reversible capacity was up to 1402.3 mAh g^−1^ after 280 charging-discharging cycles. The discharge specific capacity of NiS nanospheres reached 814.8 mAh g^−1^ at 0.8 A g^−1^, and it increased to 1130.5 mAh g^−1^ when back to 0.1 A g^−1^, indicating an enhanced rate capability. The outstanding electrochemical performance indicated that the NiS nanosphere materials are more potential anodes for LIBs.

## Methods of Preparation and Characterization

### Preparation of NiS Nanospheres

**FIGURE 1:**
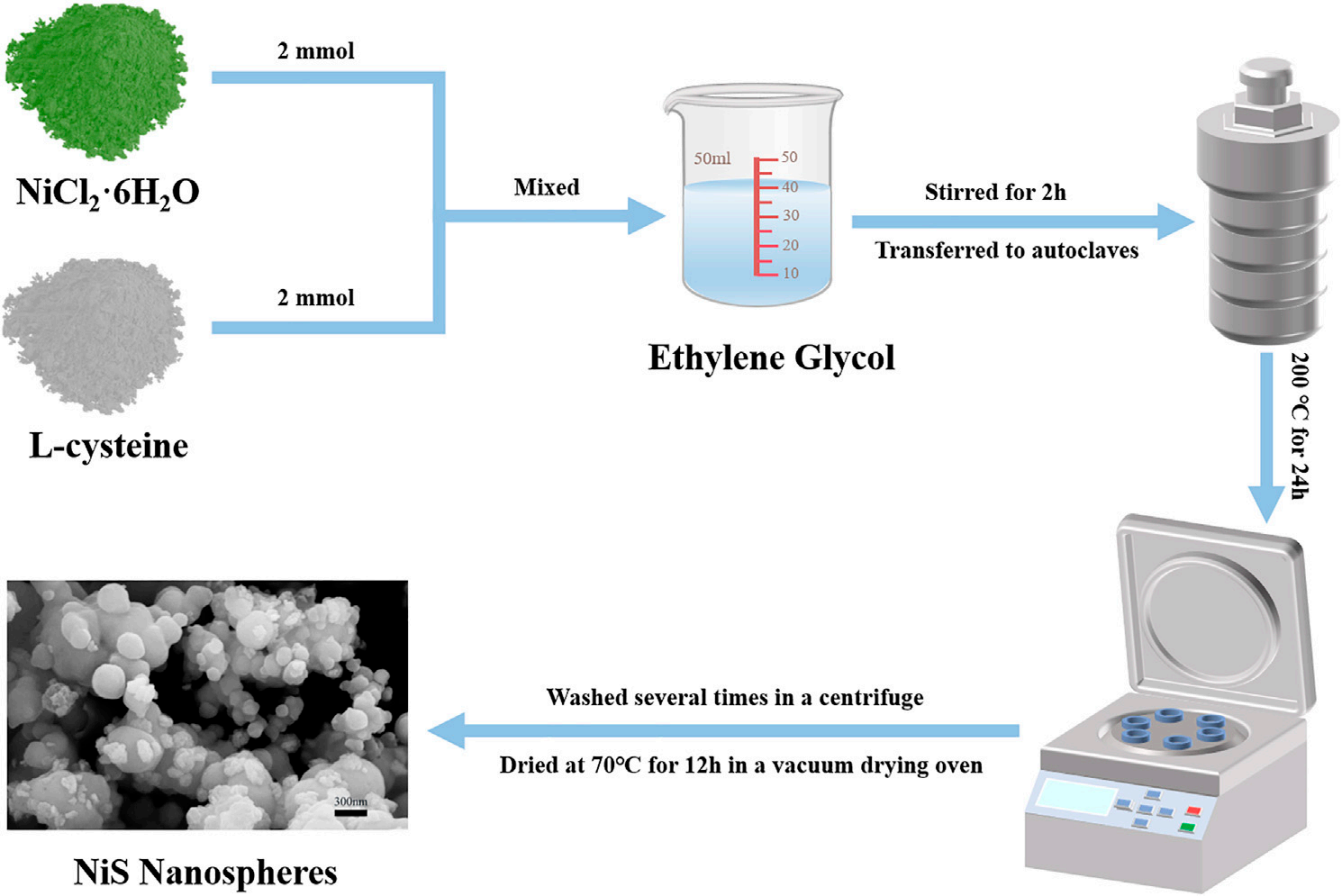
The schematic diagram of preparing NiS nanospheres.

The schematic diagram of the preparation process of NiS nanospheres is shown in Figure 1. There were 244.85 mg (2 mmol) of L-cysteine and 475.38 mg (2 mmol) of NiCl_2_·6H_2_O added into ethylene glycol (35 ml) and stirred by a magnetic stirrer for more than 2 h. The mixture was transferred to a Teflon-sealed autoclave and thoroughly reacted for 24 h at 200°C. The black powder samples were acquired after alternately washing 6 times with deionized water and ethanol in a centrifuge (10,000 rpm for 10 min) and drying at 70°C in a vacuum for 12 h.

### Characterization of NiS Nanospheres

X-ray diffraction (XRD, SmartLab, Rigku Japan) and scanning electron microscope (SEM, GeminiSEM300, Zeiss, Germany) were used to characterize the composition, structure, and morphology of the black powder. The scan rate of the Cu Кα radiation is 5°/min in the range of 20°–80° for the XRD measurements.

### Electrochemical Test of NiS Nanospheres

The anodes were made of carboxymethyl cellulose (CMC), carbon black, and NiS nanospheres powders (mass ratio 7:2:1). After coating the mixed paste on copper foil uniformly, the copper foil was dried for 12 h at 70°C under vacuum and cut into discs (113 mm^2^). The CR-2032 cells were assembled in argon with Celgard 2250 films used as the diaphragm, 1M LiPF6 solution with dimethyl ethyl carbonate and ethyl carbonate (v/v = 1:1) used as the electrolyte, and lithium disks used as counter-electrodes.

Land-CT3001A battery testing systems were used for the cycle performance test of NiS nanospheres at several different current densities. The electrochemical impedance spectroscopy (EIS, 10^−2^–10^5^ Hz) and the cyclic voltammetry (CV, 0.1–1.5 mV s^−1^) curves were determined by a CHI660E electrochemical workstation. The electrochemical tests were realized at room temperature between 0.01 and 3.0 V.

## Results and Discussion

### Structure and Morphology of NiS Nanospheres

**FIGURE 2:**
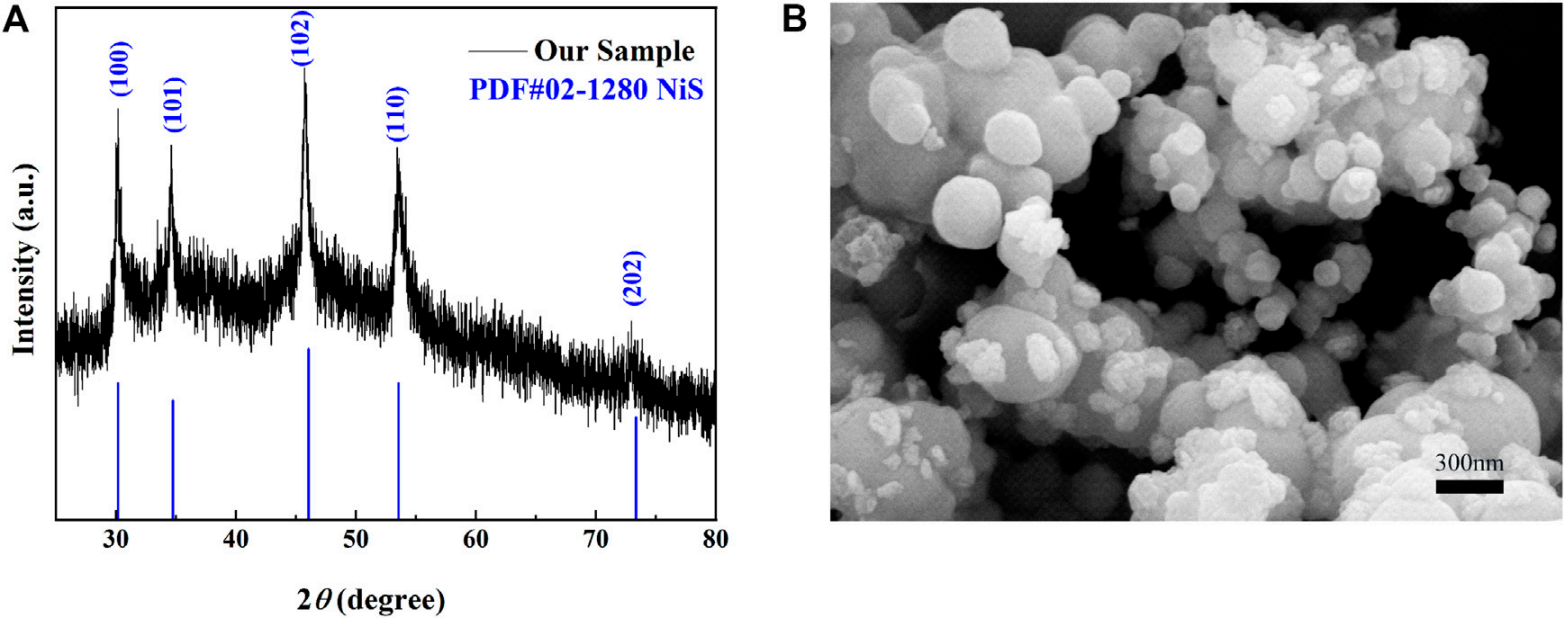
**(A)** XRD patterns and **(B)** the SEM image of the material.

The XRD patterns of the materials shown in Figure 2A have a high degree of matching with the standard card PDF No. 02-1280, which shows that the sample is pure NiS and no other components exist. The sharp diffraction peaks indicate that the samples are crystalline. In addition, the different peaks at 30.167°, 34.742°, 46.034°, 53.546°, and 73.327°, respectively, corresponded to the (100), (101), (102), (110), and (202) crystal planes of NiS. To further investigate the morphology, the NiS material was tested by SEM. The materials consisted of nanospheres with sizes between 100 and 500 nm as shown in Figure 2B.

### Electrochemical Performance of NiS Nanospheres

**FIGURE 3:**
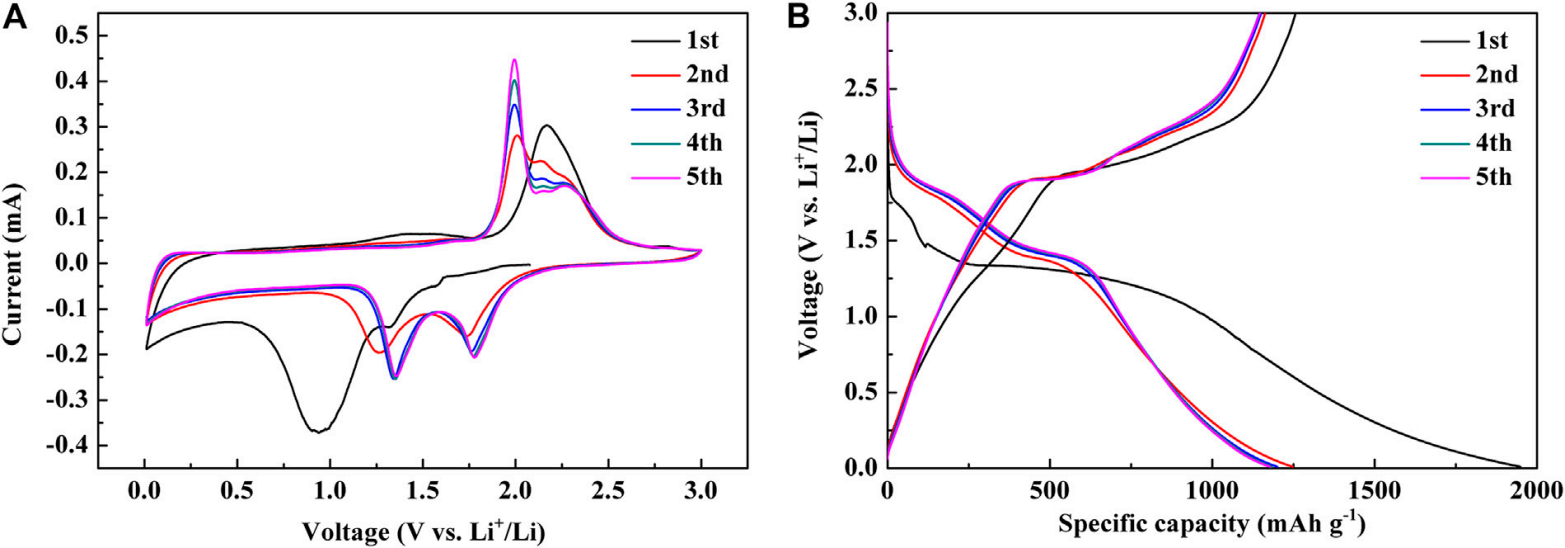
**(A)** Initial five CV curves. **(B)** Initial five discharge and charge curves.

The initial five CV curves were measured at 0.1 mV s^−1^, as is shown in Figure 3A. In CV curves, three reduction peaks existed near 0.94, 1.32, and 1.58 V during the first cathode sweep (lithiation). The peak at 0.94 V denotes the formation of the solid electrolyte interphase (SEI) layer, which can be seen from the fact that this peak no longer exists in the second circle (Duan et al., 2015; Fan et al., 2017; Jin et al., 2017; Dong et al., 2019). The reduction peaks at 1.58 and 1.32 V represent the reduction process from NiS to Ni, which corresponds to the two reactions of Equations 1 and 2, respectively (Wang et al., 2011; Dong et al., 2019; Xia et al., 2021).

$$3\mathrm{NiS}+2Li^{+}+2e^{-}\to Ni_{3}S_{2}+Li_{2}S$$ (1)

$$Ni_{3}S_{2}+4Li^{+}+4e^{-}\to 3\mathrm{Ni}+2Li_{2}S$$ (2)

In the second cycle, the two peaks shifted to 1.74 and 1.27 V, while in the third cycle, they shifted to 1.77 and 1.35 V, which was due to the activation of the materials (Ni et al., 2012). The two reduction peaks in subsequent cycles almost completely coincide with that in the third cycle, which indicates the stable reduction reaction process.

Two oxidation peaks can be observed near 1.46 and 2.17 V during the first anode sweep (delithiation). The peak at 1.46 V, which vanishes in following cycles, represents the decomposition of the SEI layer (Vadivazhagan et al., 2021; Xia et al., 2021), and the peak at 2.17 V relates to the reaction of Ni to NiS (Vadivazhagan et al., 2021), which corresponds to Equation 3 (Vadivazhagan et al., 2021).

$$\mathrm{Ni}+Li_{2}S\to \mathrm{NiS}+2Li^{+}+2e^{-}$$ (3)

The oxidation peak at 2.17 V split into three peaks at 1.99, 2.14, and 2.27 V in the second cycle, which also represents the production of NiS (Han et al., 2017; Ren et al., 2021; Xia et al., 2021). In the subsequent cycles, the peak positions of the oxidation processes almost completely coincide with that in the second cycle, which shows the stable oxidation reaction process and cycle stability.

The initial five constant current discharge-charge curves of NiS nanospheres were measured at 100 mA g^−1^, as shown in Figure 3B. There is a small platform between 1.6 and 1.7 V and a large platform between 1.0 and 1.5 V during the first discharge process. The small platform corresponds to the reduction process of NiS to Ni_3_S_2_, while the large platform signifies the formation of the SEI layer as well as the reduction process of Ni_3_S_2_ to Ni, which completely corresponds to the CV curves. There is a small platform near 1.4 V and a large platform near 2.0 V in the first charge process, which corresponds very well to the oxidation peaks in the CV curves. As shown in Figure 3B, the discharge and charge capacities of the first cycle are 1949 mAh g^−1^ and 1257 mAh g^−1^ respectively. The discharge and charge capacities can be stable around 1200 mAh g^−1^ in the following four cycles, which are much higher than the theoretical capacity of NiS (Li Q. et al., 2020; Kim et al., 2020). The phenomenon could originate from the reversible formation of polymeric gel-like films around the transition metal particles (Laruelle et al., 2002), the interface lithium storage (Zhukovskii et al., 2006), and the surface conversion of LiOH to Li_2_O and LiH (Hu et al., 2013). It is worth noting that the discharge-charge curves almost completely coincide after the first circle, which shows the great electrochemical stability and reversibility.

**FIGURE 4:**
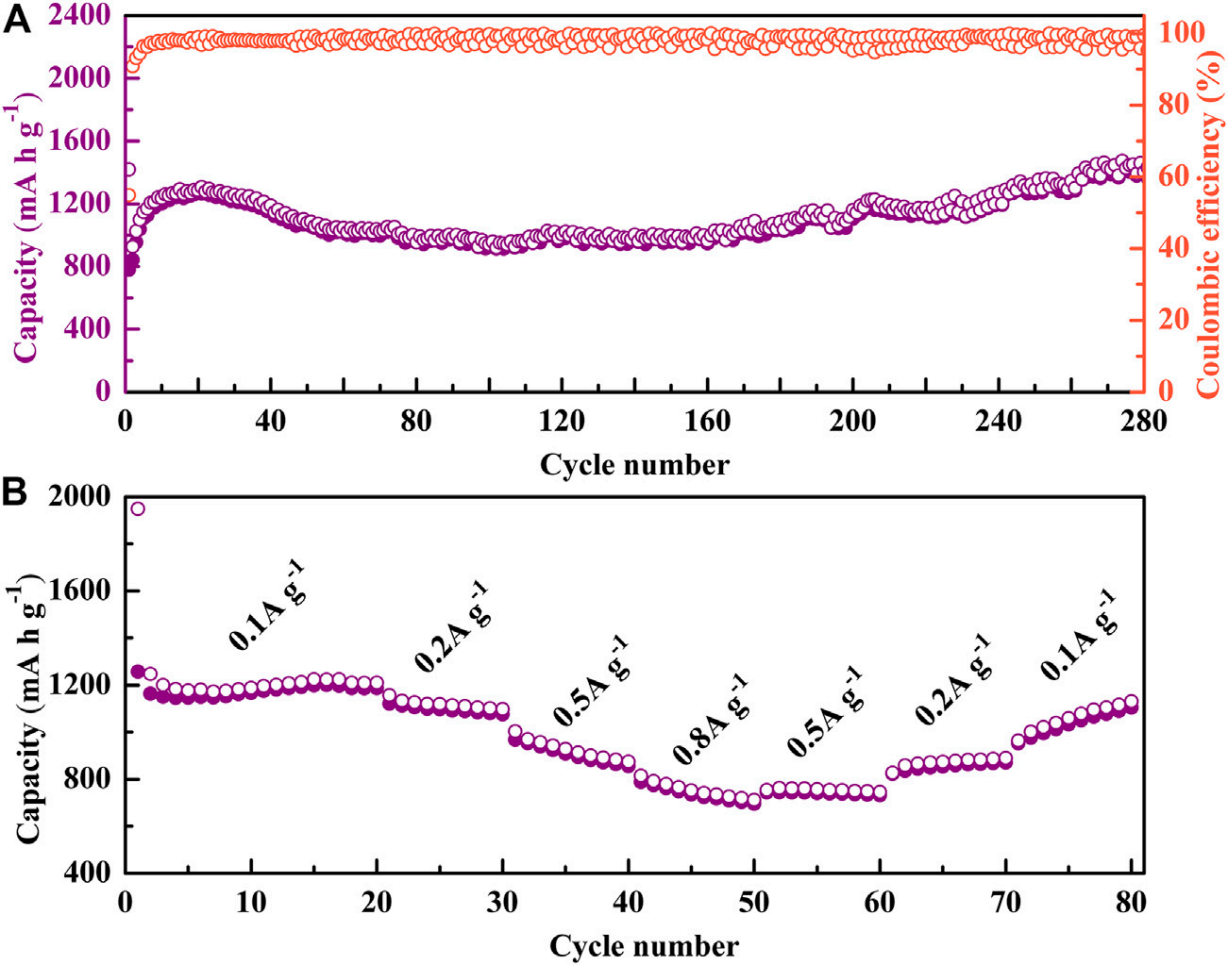
**(A)** Cycle performance of NiS nanospheres. **(B)** Rate capabilities of NiS nanospheres.

The cycle performance and the rate capabilities of NiS nanospheres were measured, as shown in Figure 4. The initial discharge and charge capacities at 200 mA g^−1^ are up to 1418.5 mAh g^−1^ and 778.3 mAh g^−1^, respectively. The initial Coulomb efficiency is 54.9% and increases rapidly to more than 90% in the second cycle, and then remains near 100% to the 280th cycle. The cycle curve has two upward trends. The first upward trend during the initial 20 cycles could be ascribed to the active process of NiS nanospheres in the first few redox reaction cycles (Li L. et al., 2019; Li S. et al., 2019), and the second upward trend during the 160th to 280th cycle could be due to the increase of active sites caused by the rupture of the NiS nanospheres during cycles (Duan et al., 2015; Wang et al., 2020, Wang L et al., 2021) and/or the reversible growth of a polymeric gel-like film resulting from the kinetically activated electrolyte degradation (Feng et al., 2013; Rui et al., 2014; Duan et al., 2015). After 280 discharge-charge cycles, the high specific capacity of 1402.3 mAh g^−1^ is obtained, which shows that the NiS nanosphere materials have good reversibility and stability as the anode of LIBs. The comparison of electrochemical properties between this work and other NiS-based electrode materials is shown in Table 1, which shows the outstanding electrochemical performance of the NiS nanospheres materials.

**TABLE 1:** Comparison of electrochemical properties between NiS_2_ nanospheres and other reported NiS-based materials.

NiS-based materials	Initial discharge capacity (mAh g^−1^)	Discharge capacity (mAh g^−1^)	Current density (mA g^−1^)	References
NiS	1418.5	1402.3 (280 cycles)	200	This work
HCNS	1132	606 (97 cycles)	100	Park and Lee (2019)
NiS@NSC	1075.4	715.9 (200 cycles)	100	Dong et al. (2019)
CSF-NiS/C	1522.8	411.6 (100 cycles)	100	Xia et al. (2021)
rGO@NiS	1520.6	1328.7 (120 cycles)	100	Ren et al. (2021)
NS@CNT	—	644 (100 cycles)	300	Fan et al. (2017)
CNTs@C@NiS	860	649 (100 cycles)	100	Jin et al. (2017)
NiS/CPC	1249	650 (50 cycles)	100	Vadivazhagan et al. (2021)
NiS@OLC	889	546 (100 cycles)	100	Han et al. (2017)
NiS/N-rGO	1240	467 (100 cycles)	0.5C	Lee et al. (2020)
NiO@*β*-NiS@Ni_3_S_2_	853.1	498.5 (100 cycles)	500	Wu et al. (2019)
NiS_2_	753	580.6 (400 cycles)	0.2C	Zhang et al. (2018)
CNF@NiS-2	1768.9	1020.6 (100 cycles)	100	Zhang et al. (2016)
NiS-PPy-CNF	806	669 (30 cycles)	100	Li et al. (2016)

It can be seen from Figure 4B that the specific capacities are 1224.3 mAh g^−1^, 1157.1 mAh g^−1^, 1003.6 mAh g^−1^, and 814.8 mAh g^−1^ at 0.1 A g^−1^, 0.2 A g^−1^, 0.5 A g^−1^, and 0.8 A g^−1^, respectively. The specific capacity returns to 1130.5 mAh g^−1^ when back to 0.1 A g^−1^, which shows that the NiS nanosphere electrodes have good reversibility and stability.

### Kinetics Characterizations of NiS Nanospheres

**FIGURE 5:**
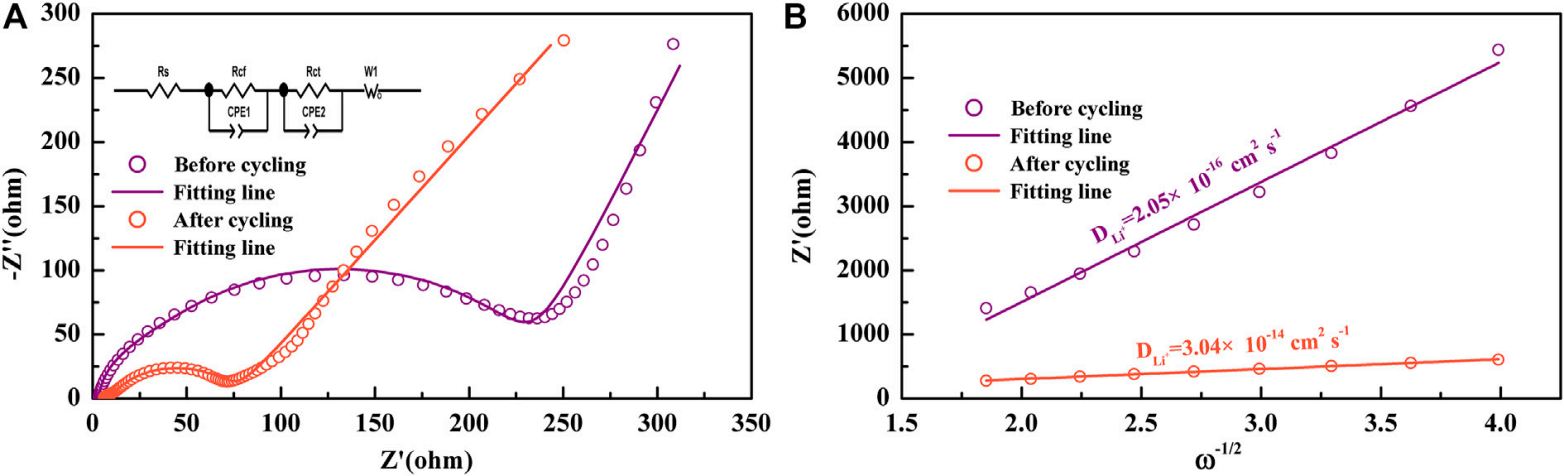
**(A)** EIS of NiS nanosphere electrodes before and after cycles; the inset is the equivalent circuit. **(B)** $Z\mathrm{’-}\omega^{\mathrm{-1}/2}$ plots in the low-frequency range of the NiS nanosphere electrodes before and after the cycling performance test.

To study the kinetic characteristics of NiS nanospheres as anodes of LIBs, the EIS of NiS nanospheres electrodes was measured before and after the cycle test, as shown in Figure 5A. The Nyquist curves are both made up of a straight line and two semicircles. The intercept denotes the resistance of the electrolyte and the electrode (*R*_s_), the diameters of the small and large semicircles denote the resistance of the SEI layer to lithium-ions migration (*R*_cf_) and the charge transfer resistance (*R*_ct_), respectively. It is obvious from Figure 5A that both the diameters before the cycle test are much larger than those after the cycle test, which shows that electrons and lithium-ions can move more easily and the NiS nanospheres electrodes have good conductivity during cycles. The insert of Figure 5A shows the equivalent circuit of the EIS curves, and all the parameters can be quantitatively fitted by it. The fitted values of *R*_s_, *R*_cf_, and *R*_ct_ are shown in Table 2. *R*_s_ changes a little before and after the cycle test, but *R*_cf_ and *R*_ct_ decrease significantly after the cycle test, which is more conducive to improving reversible capacity and cycle stability (Teng et al., 2020; Ren et al., 2021).

**TABLE 2:** Kinetics parameters of NiS nanosphere electrodes.

Parameters	Before cycling	After cycling
*R*_s_ (Ohm)	3.438	6.25
*R*_cf_ (Ohm)	193.2	64.24
*R*_ct_ (Ohm)	27.81	3.847

The diffusion coefficient of lithium-ions can be obtained by the following equations (Chen et al., 2018; Teng et al., 2020):

$$Z\prime =R_{s}+R_{\mathrm{ct}}+R_{t}+\sigma \omega^{-1/2}$$ (4)

$$D_{Li^{+}}=\frac{R^{2}T^{2}}{2A^{2}n^{4}F^{4}C^{2}\sigma^{2}}$$ (5)

where *R* represents the gas constant, *T* represents the thermodynamic temperature, here is the room temperature, *A* represents the surface area of the electrode, *n* stands for the number of electrons transferred in the oxidation or reduction reaction per molecule, *f* represents the Faraday constant, *C* represents the concentration of lithium-ions, and σ represents the slopes of $Z\prime -\omega^{-1/2}$ plots in Figure 5B. By calculation, the diffusion coefficients of lithium-ions before and after the cyclic test are 2.05 × 10^−16^ cm^2^ s^−1^ and 3.04 × 10^–14^ cm^2^ s^−1^, respectively. With the increase of ion activity, the diffusion rate of lithium-ions and electrons is faster after the cycle test, which may also be one of the reasons for the increase of reversible capacity of NiS nanospheres electrodes during cycling.

**FIGURE 6:**
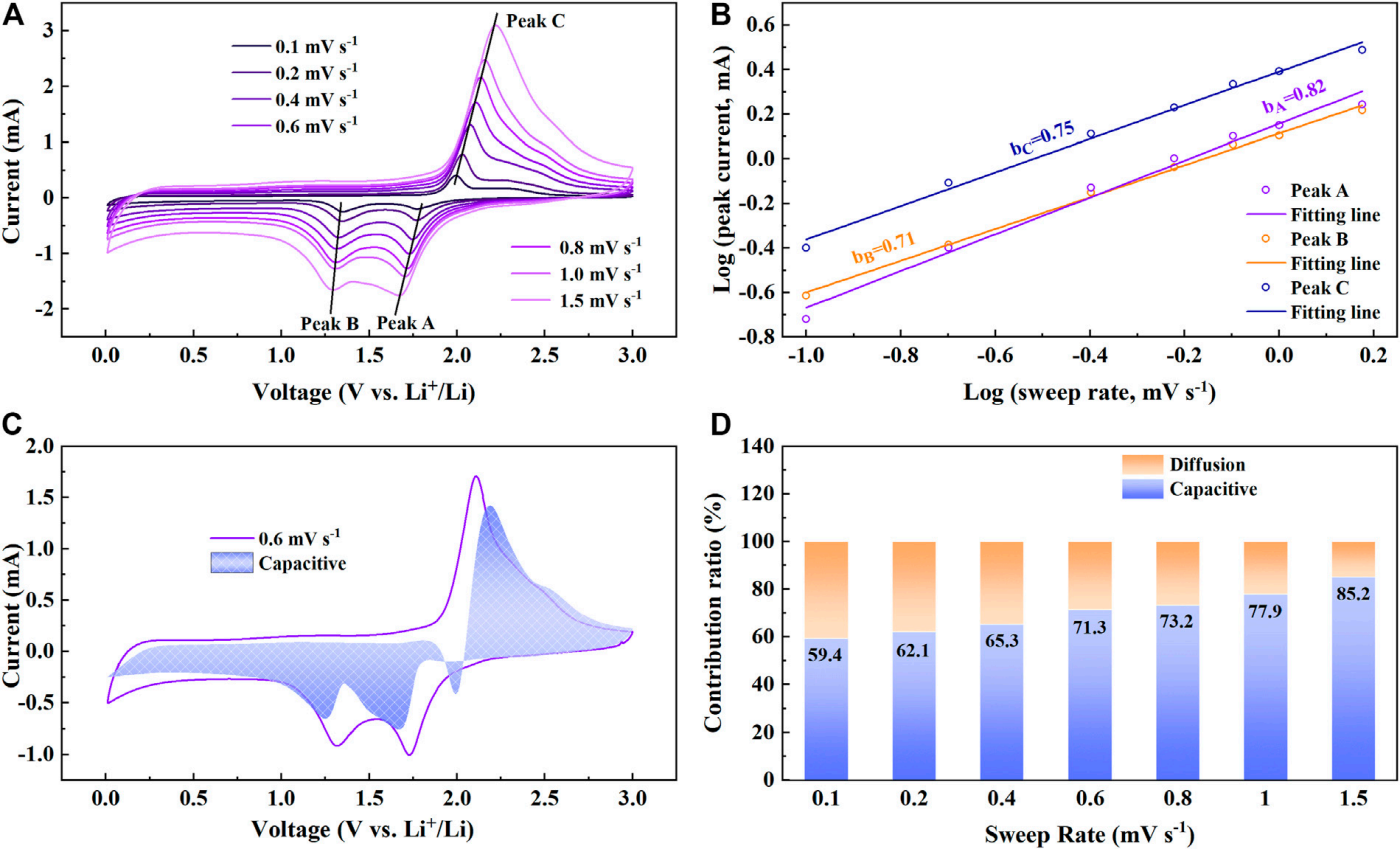
**(A)** CV curves at different sweep rates. **(B)** The corresponding plots of log(*i*) vs. log(*v*) at three redox peaks. **(C)** The CV curves and the voltage distribution (shaded part) at the sweep rate of 0.6 mV s^−1^. **(D)** Contribution ratio of capacitive at various sweep rates.

To further study the dynamic characteristics of the NiS nanospheres, CV curves at different sweep rates were measured. The shapes of CV curves in Figure 6A are very similar, only the intensities of the peaks increase with the increase of sweep rates, which shows that NiS nanosphere electrodes have good cyclic reversibility in the process of lithium and delithium (Ren et al., 2021). The contribution proportions of ion diffusion and capacitance effect in the cycles at different sweep rates can be roughly estimated by the following two equations (Ge et al., 2018; Li W. et al., 2020).

$$i=av^{b}$$ (6)

log(i)=b⁡log(v)+log(a) (7)

where *i* represents the peak current, and *v* represents the sweep rate. It indicates that the charge-discharge process is dominated by ion diffusion when *b* approaches 0.5, while when *b* approaches 1, the charge-discharge process mainly depends on the capacitance effect. The *b* values corresponding to the three peaks of redox reactions are 0.82, 0.71, and 0.75, respectively, as shown in Figure 6B, which shows that the current is the result of the joint action of ion diffusion and capacitance effect in these three reaction processes.

**FIGURE 7:**
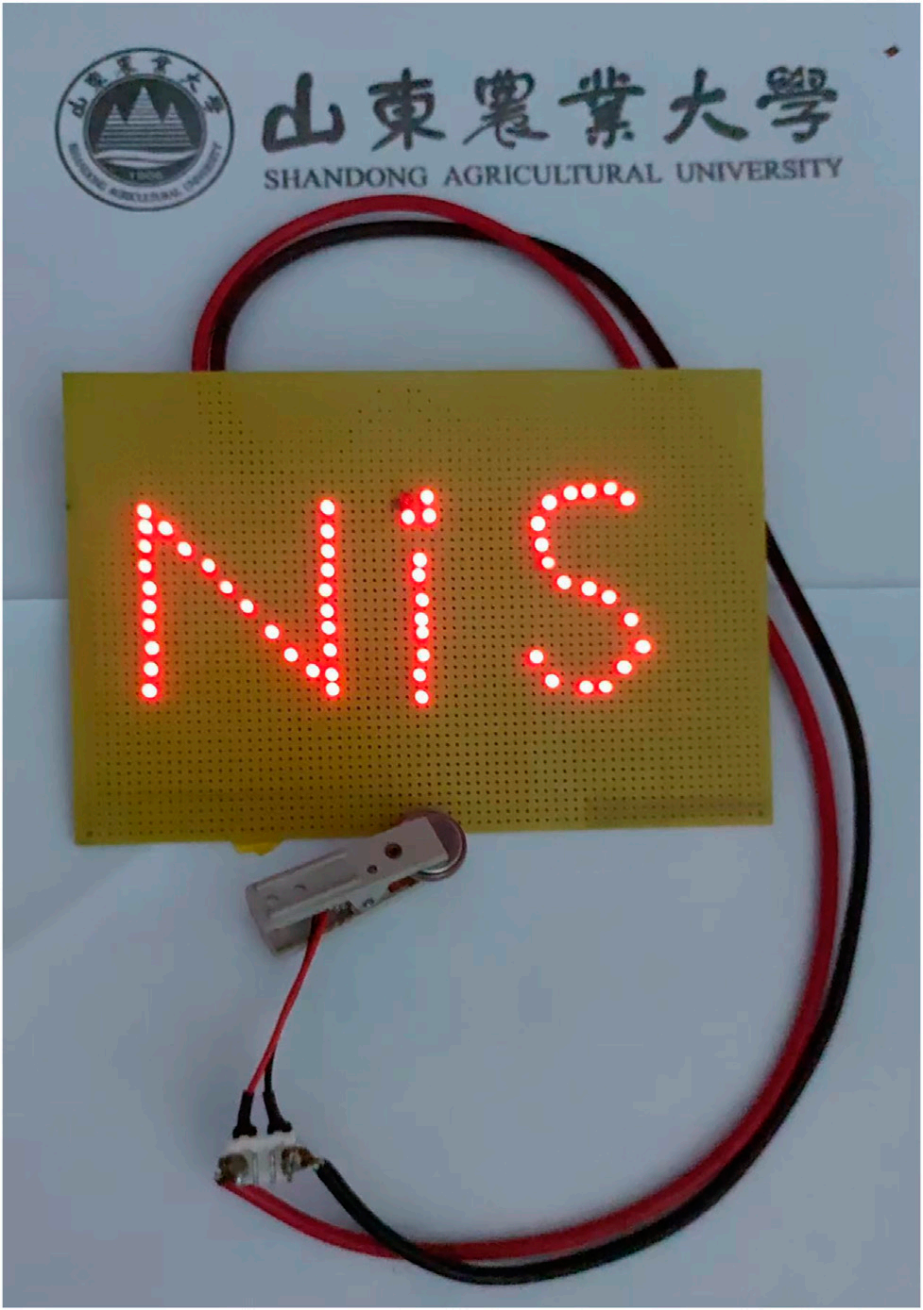
The LED lamps of “NiS” illuminated by two cells in series.

To further research the role of capacitance effect in NiS nanospheres electrodes, the following equation can be used to quantitatively calculate the current contribution proportion of capacitance effect (Wang et al., 2007; Ge et al., 2018).

$$i\left(V\right)=k_{1}v+k_{2}v^{1/2}$$ (8)

where $k_{1}v$ represents the current contribution of capacitance effect and $k_{2}v^{1/2}$ represents the current contribution of the ion diffusion. Figure 6C shows the voltage distribution (shaded part) of capacitive current at 0.6 mV s^−1^. The proportion of capacitive current is great at 0.6 mV s^−1^, reaching 71.3%. Figure 6D shows the proportions of capacitance effect and ion diffusion in current contribution at different sweep rates. The current contribution proportion of capacitance effect increases from 59.4 to 85.2% with the increases of sweep rate from 0.1 mV s^−1^ to 1.5 mV s^−1^. The large current contribution of the capacitance effect also shows that NiS nanosphere electrodes have good rate capability. Two cells in series were used to light up the LED lamps of “NiS”, as shown in Figure 7, which indicates the potential application of NiS nanosphere material as anode for LIBs.

## Conclusion

In this paper, NiS nanospheres were successfully synthesized by a simple hydrothermal method. The NiS nanosphere materials used as the anode of LIBs are not only simple to manufacture but also show outstanding electrochemical performance. After 280 charging-discharging circles, a high reversible specific capacity of 1402.3 mAh g^−1^ at 200 mA g^−1^ was obtained. In addition, the NiS nanosphere electrodes display a good rate capability. The reversible capacity still reaches 814.8 mAh g^−1^ even at 0.8 A g^−1^. In addition, the reversible capacity can still reach 1130.5 mAh g^−1^ when back to 0.1 A g^−1^. Furthermore, the conductivity after the cycle test is higher than that before the cycle test, which may be one of the reasons why the reversible capacity of NiS nanosphere electrodes increases during cycling. The proportion of capacitance contribution reaches 85.2% at 1.5 mV s^−1^, showing a strong rate capability of NiS nanospheres electrodes. The outstanding cycling stability and rate capability indicated that the NiS nanosphere materials are more promising anodes for LIBs.

## Data Availability

The raw data supporting the conclusion of this article will be made available by the authors, without undue reservation.

## References

[B1] Chen H. Zhang B. Wang X. Dong P. Tong H. Zheng J.-c. (2018). CNT-decorated Na_3_V_2_(PO_4_)_3_ Microspheres as a High-Rate and Cycle-Stable Cathode Material for Sodium Ion Batteries. ACS Appl. Mater. Inter. 10, 3590–3595. 10.1021/acsami.7b16402 29356505

[B2] Dong X. Deng Z.-P. Huo L.-H. Zhang X.-F. Gao S. (2019). Large-Scale Synthesis of NiS@N and S Co-doped Carbon Mesoporous Tubule as High Performance Anode for Lithium-Ion Battery. J. Alloys Compounds. 788, 984–992. 10.1016/j.jallcom.2019.02.326

[B3] Duan W. Yan W. Yan X. Munakata H. Jin Y. Kanamura K. (2015). Synthesis of Nanostructured Ni_3_S_2_ with Different Morphologies as Negative Electrode Materials for Lithium Ion Batteries. J. Power Sourc. 293, 706–711. 10.1016/j.jpowsour.2015.05.098

[B4] Fan P. Liu H. Liao L. Fu J. Wang Z. Lv G. (2017). Flexible and High Capacity Lithium-Ion Battery Anode Based on a Carbon Nanotube/Electrodeposited Nickel Sulfide Paper-like Composite. RSC Adv. 7, 49739–49744. 10.1039/c7ra08239h

[B5] Feng N. Hu D. Wang P. Sun X. Li X. He D. (2013). Growth of Nanostructured Nickel Sulfide Films on Ni Foam as High-Performance Cathodes for Lithium Ion Batteries. Phys. Chem. Chem. Phys. 15, 9924–9930. 10.1039/c3cp50615k 23673428

[B6] Gao M. Zhou W.-Y. Mo Y.-X. Sheng T. Deng Y. Chen L. (2021). Outstanding Long-Cycling Lithium−Sulfur Batteries by Core-Shell Structure of S@Pt Composite with Ultrahigh Sulfur Content. Adv. Powder Mater. 10.1016/j.apmate.2021.09.006

[B7] Ge P. Hou H. Li S. Huang L. Ji X. (2018). Three-Dimensional Hierarchical Framework Assembled by Cobblestone-Like CoSe_2_@C Nanospheres for Ultrastable Sodium-Ion Storage. ACS Appl. Mater. Inter. 10, 14716–14726. 10.1021/acsami.8b01888 29635915

[B8] Gu Z. Y. Guo J. Z. Zhao X. X. Wang X. T. Xie D. Sun Z. H. (2021). High‐ionicity Fluorophosphate Lattice via Aliovalent Substitution as Advanced Cathode Materials in Sodium‐ion Batteries. InfoMat. 3, 694–704. 10.1002/inf2.12184

[B9] Han D. Xiao N. Liu B. Song G. Ding J. (2017). One-pot Synthesis of Core/Shell-Structured NiS@onion-Like Carbon Nanocapsule as a High-Performance Anode Material for Lithium-Ion Batteries. Mater. Lett. 196, 119–122. 10.1016/j.matlet.2017.03.042

[B10] Hong S.-H. Song M. Y. (2018). Syntheses of Nano-Sized Co-Based Powders by Carbothermal Reduction for Anode Materials of Lithium Ion Batteries. Ceramics Int. 44, 4225–4229. 10.1016/j.ceramint.2017.12.002

[B11] Hou X. Li W. Wang Y. Li S. Meng Y. Yu H. (2020). Sodium-Based Dual-Ion Batteries via Coupling High-Capacity Selenium/Graphene Anode with High-Voltage Graphite Cathode. Chin. Chem. Lett. 31, 2314–2318. 10.1016/j.cclet.2020.04.021

[B12] Hu Y.-Y. Liu Z. Nam K.-W. Borkiewicz O. J. Cheng J. Hua X. (2013). Origin of Additional Capacities in Metal Oxide Lithium-Ion Battery Electrodes. Nat. Mater. 12, 1130–1136. 10.1038/nmat3784 24185759

[B13] Jin R. Jiang Y. Li G. Meng Y. (2017). Amorphous Carbon Coated Multiwalled Carbon Nanotubes@transition Metal Sulfides Composites as High Performance Anode Materials for Lithium Ion Batteries. Electrochimica Acta. 257, 20–30. 10.1016/j.electacta.2017.10.078

[B14] Kim H. Choi W. Yoon J. Um J. H. Lee W. Kim J. (2020). Exploring Anomalous Charge Storage in Anode Materials for Next-Generation Li Rechargeable Batteries. Chem. Rev. 120, 6934–6976. 10.1021/acs.chemrev.9b00618 32101429

[B15] Laruelle S. Grugeon S. Poizot P. Dollé M. Dupont L. Tarascon J.-M. (2002). On the Origin of the Extra Electrochemical Capacity Displayed by MO/Li Cells at Low Potential. J. Electrochem. Soc. 149, A627–A634. 10.1149/1.1467947

[B16] Lee Y.-J. Ha T.-H. Cho G.-B. Kim K.-W. Ahn J.-H. Cho K.-K. (2020). Fabrication of Nickel Sulfide/Nitrogen-Doped Reduced Graphene Oxide Nanocomposite as Anode Material for Lithium-Ion Batteries and its Electrochemical Performance. J. Nanosci. Nanotechnol. 20, 6782–6787. 10.1166/jnn.2020.18783 32604513

[B17] Li H. Guo S. Shin K. Wong M. S. Henkelman G. (2019). Design of a Pd-Au Nitrite Reduction Catalyst by Identifying and Optimizing Active Ensembles. ACS Catal. 9, 7957–7966. 10.1021/acscatal.9b02182

[B18] Li L. Wang L. Zhang M. Huang Q. Chen L. Wu F. (2019). High-Performance Lithium-Ion Battery Anodes Based on Mn_3_O_4_/Nitrogen-Doped Porous Carbon Hybrid Structures. J. Alloys Compounds. 775, 51–58. 10.1016/j.jallcom.2018.10.106

[B19] Li S. Li B. Zhong Y. Pan Z. Xu M. Qiu Y. (2019). Mn_2_O_3_@C Yolk-Shell Nanocubes as Lithium-Storage Anode with Suppressed Surface Electrolyte Decomposition. Mater. Chem. Phys. 222, 256–262. 10.1016/j.matchemphys.2018.10.015

[B20] Li H. Hu Z. Xia Q. Zhang H. Li Z. Wang H. (2021). Operando Magnetometry Probing the Charge Storage Mechanism of CoO Lithium‐Ion Batteries. Adv. Mater. 33, 2006629. 10.1002/adma.202006629 33576103

[B21] Li S. Gu Z.-Y. Guo J.-Z. Hou X.-K. Yang X. Zhao B. (2021). Enhanced Electrode Kinetics and Electrochemical Properties of Low-Cost NaFe_2_PO_4_(SO_4_)_2_ via Ca^2+^ Doping as Cathode Material for Sodium-Ion Batteries. J. Mater. Sci. Technology. 78, 176–182. 10.1016/j.jmst.2020.10.047

[B22] Li Z. Zhang Y. Li X. Gu F. Zhang L. Liu H. (2021). Reacquainting the Electrochemical Conversion Mechanism of FeS2 Sodium-Ion Batteries by Operando Magnetometry. J. Am. Chem. Soc. 143, 12800–12808. 10.1021/jacs.1c06115 34369752

[B23] Li Q. Li H. Xia Q. Hu Z. Zhu Y. Yan S. (2020). Extra Storage Capacity in Transition Metal Oxide Lithium-Ion Batteries Revealed by *In Situ* Magnetometry. Nat. Mater. 20, 76–83. 10.1038/s41563-020-0756-y 32807921

[B24] Li W Liang H.-J. Hou X.-K. Gu Z.-Y. Zhao X.-X. Guo J.-Z. (2020). Feasible Engineering of Cathode Electrolyte Interphase Enables the Profoundly Improved Electrochemical Properties in Dual-Ion Battery. J. Energ. Chem. 50, 416–423. 10.1016/j.jechem.2020.03.043

[B25] Li X. Chen Y. Zou J. Zeng X. Zhou L. Huang H. (2016). Stable Freestanding Li-Ion Battery Cathodes by *In Situ* Conformal Coating of Conducting Polypyrrole on NiS-Carbon Nanofiber Films. J. Power Sourc. 331, 360–365. 10.1016/j.jpowsour.2016.09.067

[B26] Liang H. Zhang H. Zhao L. Chen Z. Huang C. Zhang C. (2022). Layered Fe_2_(MoO_4_)_3_ Assemblies with Pseudocapacitive Properties as Advanced Materials for High-Performance Sodium-Ion Capacitors. Chem. Eng. J. 427, 131481–131489. 10.1016/j.cej.2021.131481

[B27] Ni S. Yang X. Li T. (2012). Fabrication of a Porous NiS/Ni Nanostructured Electrode via a Dry Thermal Sulfuration Method and its Application in a Lithium Ion Battery. J. Mater. Chem. 22, 2395–2397. 10.1039/c2jm15394g

[B28] Park J. H. Lee J. W. (2019). Visualized Pulverization via *Ex Situ* Analyses: Nickel Sulfide Anode Caged in a Hierarchical Carbon. J. Electrochem. Soc. 166, A838–A847. 10.1149/2.1071904jes

[B29] Ren H. Wang J. Cao Y. Luo W. Sun Y. (2021). Nickel Sulfide Nanoparticle Anchored Reduced Graphene Oxide with Improved Lithium Storage Properties. Mater. Res. Bull. 133, 111047–111057. 10.1016/j.materresbull.2020.111047

[B30] Rui X. Tan H. Yan Q. (2014). Nanostructured Metal Sulfides for Energy Storage. Nanoscale. 6, 9889–9924. 10.1039/c4nr03057e 25073046

[B31] Teng X. Zhang F. Li Q. Wang X. Ye W. Li H. (2020). Interfacial Engineering of Self-Supported SnO_2_ Nanorod Arrays as Anode for Flexible Lithium-Ion Batteries. J. Electrochem. Soc. 167, 120515–120524. 10.1149/1945-7111/abac86

[B32] Vadivazhagan M. Shakkeel N. K. Nallathamby K. (2021). Demonstration of Biocarbon-Added NiS Porous Nanospheres as a Potential Anode for Lithium-Ion Batteries. Energy Fuels. 35, 8991–9000. 10.1021/acs.energyfuels.1c00582

[B33] Wang J. Polleux J. Lim J. Dunn B. (2007). Pseudocapacitive Contributions to Electrochemical Energy Storage in TiO_2_ (Anatase) Nanoparticles. J. Phys. Chem. C. 111, 14925–14931. 10.1021/jp074464w

[B34] Wang K. Li L. Zhang T. Liu Z. (2014). Nitrogen-doped Graphene for Supercapacitor with Long-Term Electrochemical Stability. Energy. 70, 612–617. 10.1016/j.energy.2014.04.034

[B35] Wang L.-H. Dai Y.-K. Qin Y.-F. Chen J. Zhou E.-L. Li Q. (2020). One-Pot Synthesis and High Electrochemical Performance of CuS/Cu_1.8_S Nanocomposites as Anodes for Lithium-Ion Batteries. Materials. 13, 3797–3808. 10.3390/ma13173797 PMC7503719 32872089

[B36] Wang L Teng X.-L. Qin Y.-F. Li Q. (2021). High Electrochemical Performance and Structural Stability of CoO Nanosheets/CoO Film as Self-Supported Anodes for Lithium-Ion Batteries. Ceramics Int. 47, 5739–5746. 10.1016/j.ceramint.2020.10.160

[B37] Wang X. Liu W. Wang C. Zhang S. Ding M. Xu X. (2021). Enhanced Formaldehyde Gas Sensing Performance of Ternary CuBi_2_O_4_ Oxides through Oxygen Vacancy Manipulation and Surface Platinum Decoration. Sensors Actuators B: Chem. 344, 130190–130199. 10.1016/j.snb.2021.130190

[B38] Wang X. Liu W. Wang T. Zhao Y. Zhao G. Zhang S. (2022). Synthesis of Multishelled SnOx/Co_3_O_4_ Amorphous/Crystalline Heterophase with Galvanic Replacement Reaction for superior HCHO Sensing. Sensors Actuators B: Chem. 350, 130876–130886. 10.1016/j.snb.2021.130876

[B39] Wang X. Zhang S. Shao M. Huang J. Deng X. Hou P. (2017). Fabrication of ZnO/ZnFe_2_O_4_ Hollow Nanocages Through Metal Organic Frameworks Route with Enhanced Gas Sensing Properties. Sensors Actuators B: Chem. 251, 27–33. 10.1016/j.snb.2017.04.114

[B40] Wang Y. Zhu Q. Tao L. Su X. (2011). Controlled-Synthesis of NiS Hierarchical Hollow Microspheres with Different Building Blocks and Their Application in Lithium Batteries. J. Mater. Chem. 21, 9248–9254. 10.1039/c1jm10271k

[B41] Wu X. Li S. Xu Y. Wang B. Liu J. Yu M. (2019). Hierarchical Heterostructures of NiO Nanosheet Arrays Grown on pine Twig-like β-NiS@Ni_3_S_2_ Frameworks as Free-Standing Integrated Anode for High-Performance Lithium-Ion Batteries. Chem. Eng. J. 356, 245–254. 10.1016/j.cej.2018.08.187

[B42] Xia G. Li X. He J. Wang Y. Gu Y. Liu L. (2021). A Biomass-Derived Biochar-Supported NiS/C Anode Material for Lithium-Ion Batteries. Ceramics Int. 47, 20948–20955. 10.1016/j.ceramint.2021.04.093

[B43] Yang Z. Y. Yuan Y. F. Zhu M. Yin S. M. Cheng J. P. Guo S. Y. (2021). Superior Rate-Capability and Long-Lifespan Carbon Nanotube-In-nanotube@Sb_2_S_3_ Anode for Lithium-Ion Storage. J. Mater. Chem. A. 9, 22334–22346. 10.1039/d1ta06708g

[B44] Zhang L. Huang Y. Zhang Y. Gu H. Fan W. Liu T. (2016). Flexible Electrospun Carbon Nanofiber@NiS Core/Sheath Hybrid Membranes as Binder-free Anodes for Highly Reversible Lithium Storage. Adv. Mater. Inter. 3, 1500467–1500476. 10.1002/admi.201500467

[B45] Zhang Y. Lu F. Pan L. Xu Y. Yang Y. Bando Y. (2018). Improved Cycling Stability of NiS_2_ Cathodes Through Designing a "Kiwano" Hollow Structure. J. Mater. Chem. A. 6, 11978–11984. 10.1039/c8ta01551a

[B46] Zhao J. Zhang Y. Wang Y. Li H. Peng Y. (2018). The Application of Nanostructured Transition Metal Sulfides as Anodes for Lithium Ion Batteries. J. Energ. Chem. 27, 1536–1554. 10.1016/j.jechem.2018.01.009

[B47] Zhao W. C. Yuan Y. F. Du P. F. Yin S. M. Guo S. Y. (2021). Intimately Coupled MoP Nanocrystalline@carbon Nanosheets-Assembled Hierarchical Mesoporous Nanospheres for High-Performance Sodium-Ion Storage. Electrochimica Acta. 389, 138712. 10.1016/j.electacta.2021.138712

[B48] Zheng Y. Q. Yuan Y. F. Tong Z. W. Yin H. Yin S. M. Guo S. Y. (2020). Watermelon-like TiO_2_ Nanoparticle (P25)@microporous Amorphous Carbon Sphere with Excellent Rate Capability and Cycling Performance for Lithium-Ion Batteries. Nanotechnology. 31, 215407. 10.1088/1361-6528/ab73be 32032007

[B49] Zhou Y. Huang Y. Pang J. Wang K. (2019). Remaining Useful Life Prediction for Supercapacitor Based on Long Short-Term Memory Neural Network. J. Power Sourc. 440, 227149–227157. 10.1016/j.jpowsour.2019.227149

[B50] Zhukovskii Y. F. Balaya P. Kotomin E. A. Maier J. (2006). Evidence for Interfacial-Storage Anomaly in Nanocomposites for Lithium Batteries from First-Principles Simulations. Phys. Rev. Lett. 96, 058302. 10.1103/PhysRevLett.96.058302 16487002

